# Effect of Co Substitution on Crystallization and Magnetic Behavior of Fe_85.45−x_Co_x_Cu_0.55_B_14_ Metallic Glass

**DOI:** 10.3390/ma13040919

**Published:** 2020-02-19

**Authors:** Lukasz Hawelek, Tymon Warski, Patryk Wlodarczyk, Marcin Polak, Przemyslaw Zackiewicz, Adrian Radon, Anna Wojcik, Aleksandra Kolano-Burian

**Affiliations:** 1 Lukasiewicz Research Network—Institute of Non-Ferrous Metals, 44-100 Gliwice, Poland; tymon.warski@imn.gliwice.pl (T.W.); patryk.wlodarczyk@imn.gliwice.pl (P.W.); marcin.polak@imn.gliwice.pl (M.P.); przemyslaw.zackiewicz@imn.gliwice.pl (P.Z.); adrian.radon@imn.gliwice.pl (A.R.); olak@imn.gliwice.pl (A.K.-B.); 2 Institute of Metallurgy and Materials Science Polish Academy of Sciences, 30-059 Krakow, Poland; wojcik.a@imim.pl

**Keywords:** soft magnetic materials, metallic glass, crystallization, magnetic properties

## Abstract

The effects of Co for Fe substitution on magnetic properties, thermal stability and crystal structure of Fe_85.45−x_Co_x_Cu_0.55_B_14_ (x = 0, 2.5, 5, 7.5, 10) melt spun amorphous alloys were investigated. The Cu content was firstly optimized to minimize the energy of amorphous phase formation by the use of a thermodynamic approach. The formation of crystalline α-Fe type phase has been described using differential scanning calorimetry, X-ray diffractometry and transmission electron microscopy. The classical heat treatment process (with heating rate 10 °C/min) in vacuum for wound toroidal cores was optimized in the temperature range from 280 to 430 °C in order to obtain the best magnetic properties (magnetic saturation Bs and coercivity Hc obtained from the B(H) dependencies) at 50 Hz frequency. For optimal heat-treated samples, the complex magnetic permeability in the frequencies 10^4^–10^8^ Hz at room temperature was measured. Finally, magnetic core losses were obtained for 1 T/50 Hz and 1.5 T/50 Hz values for samples annealed at T = 310 °C. An analysis of transmission electron microscope images and electron diffraction patterns confirmed that high magnetic parameters are related to the coexistence of the amorphous and nanocrystalline phases.

## 1. Introduction

The demand for efficient materials for power electronic applications, owing the superior soft magnetic properties and saturation magnetic induction, has been growing for many years. Although the FINEMET type alloys possess high maximum relative permeability µ_max_ = 5 × 10^5^, they also have poor saturation magnetic induction (Bs = 1.24 T), which limits their industrial application [1,2,3]. On the other hand, NANOPERM alloys have enhanced saturation magnetization, but their soft magnetic properties are deteriorated (µ_max_ = 5 × 10^4^, Bs = 1.52 T) [1]. As it was previously shown, Si has a detrimental effect on saturation magnetization in Co content alloys due to the formation of Fe_3_Si-type phase [4]. It was also shown by Ohta and Yoshizawa in [5,6] that there is possible development of magnetic materials (Fe-Cu-B and Fe-Cu-Si-B) with a high magnetic saturation up to 1.8 T and relatively low coercivity Hc. They show that amount of Cu should be increased together with the Si content and for Si = 4 at.% optimal chemical composition is Fe_80.5_Cu_1.5_Si_4_B_14_. Moreover, for Fe-Si-B-Nb-Cu alloys Ohnuma shown that the kinetics for Cu clustering varies depending on the Cu content, by which the final grain size of Fe–Si crystal is influenced [7].

In the present study, we proposed firstly a thermodynamic approach to optimize Cu content in the binary Fe_86_B_14_ alloy in the context of minimum amorphization energy. Then, the effect of varying Co content on magnetic properties, Bs, Hc and magnetic permeability, was investigated. The aim of this study was to optimize the alloy composition and annealing treatment to obtain satisfactory magnetic properties.

## 2. Thermodynamic Approach

**Figure 1:**
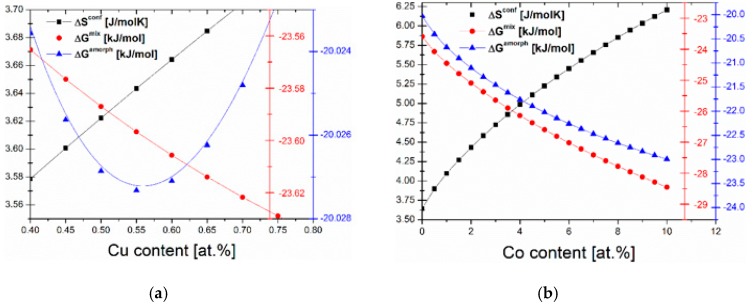
The thermodynamic parameters dependences in the function of Cu (Fe_86−y_Cu_y_B_14_) (**a**) and Co (Fe_8__5.45−x_Co_x_Cu_0.55_B_14_) (**b**) content.

The optimal content of Cu in the sense of minimum value of amorphous phase formation energy, was determined on the basis of optimization of thermodynamic parameters. Three different parameters, i.e., configurational entropy (Δ*S^config^*), Gibbs free energy of mixing (Δ*G^mix^*) and Gibbs free energy of amorphous phase formation (Δ*G^amorph^*) were calculated for different copper contents in Fe_86−y_Cu_y_B_14_ alloys [8]. The analysis results are presented in Figure 1. It can be noticed, that with the increasing copper content, the configurational entropy increases, however, the Δ*G^mix^* decreases. The changes in Gibbs free energy of mixing can be negligible, which is associated with the positive enthalpy of mixing of binary Fe-Cu system (13 kJ/mol). As can be seen, the changes in the Δ*G^amorph^* are different and minimum can be observed when Cu content is equal to 0.55 at.%. This can be related to the balance between configurational entropy and enthalpy of formation of amorphous phase in this system. According to that, this Cu content was marked as optimal of amorphous phase formation energy and further calculations were performed for Fe_85.45_Cu_0.55_B_14_ alloys. It is a well known fact that the small cobalt addition enhances the magnetic saturation and deteriorates the magnetic permeability of amorphous and nanocrystalline alloys [9,10,11,12]. Therefore, the same procedure was applied to determine the influence of Co content on the thermodynamic properties of Fe_85.45−x_Co_x_Cu_0.55_B_14_ alloys. The same tendency as for copper was observed in the case of Δ*S^config^* and Δ*G^mix^*; however, introduction of the higher content of Co results in decreasing Δ*G^amorph^*. Therefore, the alloys with higher Co content and Cu content equal to 0.55 should be characterized by a higher glass forming ability.

## 3. Materials and Methods

Amorphous alloys with nominal compositions of Fe_85.45−x_Co_x_Cu_0.55_B_14_ (x = 0, 2.5, 5, 7.5, 10) in the form of ribbons with a 20–28 µm thickness and 6–7 mm width were obtained by melt spinning technique (at 30 m/s Cu wheel speed). To achieve the optimal magnetic parameters, the toroidal cores were isothermally annealed for 20 min in vacuum furnace (5 × 10^−4^ mbar) at different temperatures, i.e., from 280 to 430 °C. Amorphousness of the as-spun and annealed ribbons was confirmed by X-ray diffraction (XRD) at room temperature using a Rigaku MiniFlex 600 diffractometer (Cu*K_α_* radiation, Tokyo, Japan). The crystallization processes were monitored by differential scanning calorimetry (DSC) with a heating rate of 10 °C/min using thermal analyzer Netzsch DSC 214 Polyma (Selb, Germany). The transmission electron microscopy (TEM) images in the bright-field (BF) mode and selected area diffraction patterns (SADPs) were recorded using Tecnai G2 F20 (200 kV, Waltham, MA, USA) electron microscope. The Remacomp C-1200 (MAGNET-PHYSIK Dr. Steingroever GmbH, Köln, Germany) magnetic measurement system was used to determine B(H), then for optimal annealed samples the magnetic core losses Ps at 1 T/50 Hz (P_10/50_) and 1.5 T/50 Hz (P_15/50_) were obtained. The complex magnetic permeability in the frequency range f = 10^4^–10^8^ Hz at room temperature of the toroidal cores was measured using the impedance analyzer Agilent 4294A (Santa Clara, CA, USA).

## 4. Results

**Figure 2:**
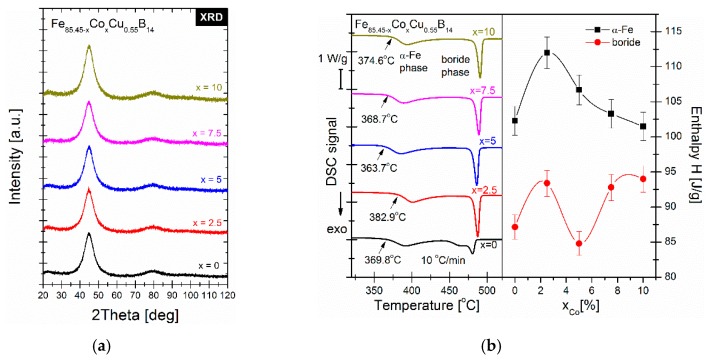
XRD patterns (**a**) and DSC signals together with enthalpy from Co content dependence (**b**) for as-spun metallic glasses.

The X-ray diffraction patterns presented in Figure 2a of as-spun ribbons show only broad amorphous halos that prove the amorphous state of all melt spun alloys. The crystallization temperatures of alloys are marked on DSC thermograms in Figure 2b. The onset of primary crystallization temperatures Tx1 of α-Fe phase for Co-free alloy equals 369.8 °C and fluctuates for Co content alloys. Firstly, increases up to 382.9 °C for Co = 2.5% then decreases to 363.7 °C for Co = 5% and slowly increases for alloys containing 7.5% and 10% of cobalt. 

**Figure 3:**
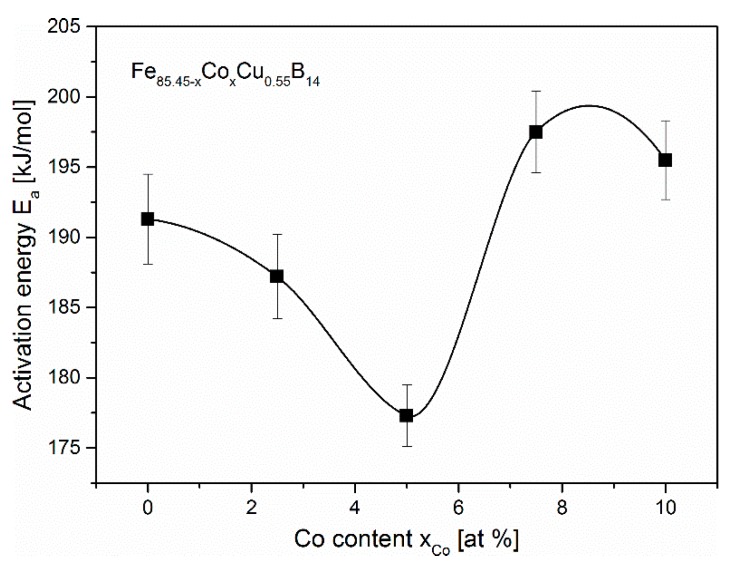
Activation energy Ea of α-Fe phase crystallization in the function of Co content. The plotted line is a guide for the eye.

On the right panel of Figure 2b, enthalpies of crystallization of both phases, i.e., α-Fe and boride are shown. The enthalpy of crystallization of boride phase varies with the cobalt content between 85 and 95 J/g. Much more interesting is the anomalous behavior of crystallization enthalpy of α-Fe phase. The maximum of crystallization enthalpy of α-Fe phase equal to 112.5 J/g has been observed for Co = 2.5%. For higher cobalt amounts enthalpy decreases slowly. Similar characteristics of enthalpy as a function of cobalt content was observed in the work by Kolano-Burian et al., where firstly, for increasing Co content, enthalpy is increasing while for farther Co substitution, enthalpy is decreasing [13]. The kinetics of α-Fe type phase crystallization has been studied by means of Differential Scanning Calorimetry (DSC) by performing heating runs with rates from 5 to 50 °C/min. For such non-isothermal crystallization process, the Kissinger model [14] was used in order to determine the average activation energies. This method is based on the equation:

$$ln\left(\frac{\phi }{T_{p}^{2}}\right)=ln\left(\frac{A_{0}R}{E_{a}}\right)-\frac{E_{a}}{\left(RT_{p}\right)}$$

where ϕ is a heating rate, $T_{p}$—temperature of the crystallization peak, $E_{a}$—activation energy, R—gas constant and $A_{0}$—pre-exponential factor. By linear fitting of $ln\left(\phi /T_{p}^{2}\right)$ vs. $1/T_{p}$ curves the average activation energy *E_a_* of the process can be determined from the slopes of these curves. The calculated *E_a_* values for the ribbons in the function of Co content are gathered in Figure 3. For the Co-free ribbon, average activation energy is equal to 191 kJ/mol. Adding cobalt to the ribbon up to 5% reduces activation energy to 177 kJ/mol (Co = 5.0%). When the cobalt content reaches 7.5%, activation energy jumps to 197 kJ/mol. Therefore, there is a minimum of activation energy for the composition with cobalt content Co = 5% at. Moreover, for this composition (Co = 5%), there is also a minimum value of α-Fe onset temperature Tx1 of crystallization (Figure 2b). 

**Figure 4:**
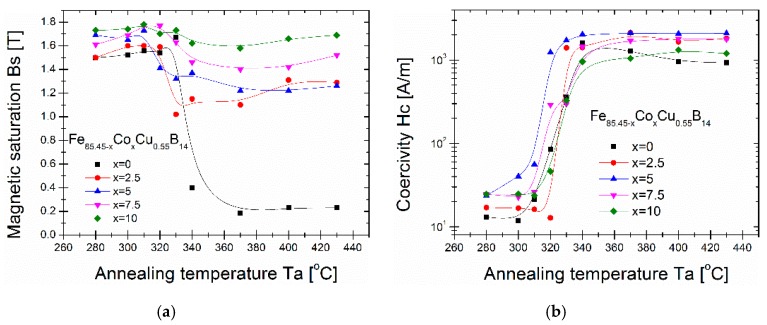
Bs (**a**) and Hc (**b**) from Ta dependences.

**Figure 5:**
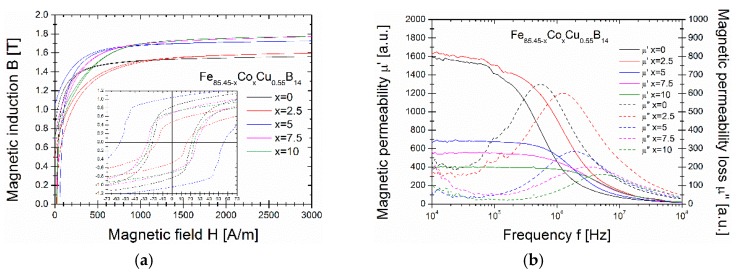
B First quarters of B(H) curves for annealed samples at 310 °C (**a**). Magnetic permeability µ’ and magnetic permeability loss µ” dependence in the function of frequency 10^4^–10^8^ Hz for annealed samples at 310 °C (**b**).

In Figure 4, the annealing temperature Ta dependences of the magnetic saturation Bs and the coercivity Hc taken from the hysteresis loops measured up to 3000 A/m for melt-spun Fe_85.45−x_Co_x_Cu_0.55_B_14_ alloys are shown. The Ta dependence of Bs for all the cobalt content alloys prove, that the Ta limitations of the Bs value decreases just above the temperature 310–320 °C. The Co content markedly enhance the Bs value from 1.55 T for Co-free alloy up to 1.79 T for Co = 10%. For samples annealed at 340 °C and higher temperatures the disproportion of the Bs value is much stronger and for Co-free alloy Bs falls to 0.2 T, while for alloys with Co, it is always higher than 1 T. For Co = 10% Bs value is always higher than 1.6 T even for Ta = 430 °C, where boride phase coexists. A very similar situation is in Ta dependence of Hc, where in the temperature range of 310–320 °C, the significant decrease in Hc values is clearly seen. The minimum value of Hc is for Co-free alloy and equals 12 A/m, while for Co = 5% it is above 30 A/m. For Co = 7.5% and Co = 10% the minimum value of Hc is equal to 25 A/m. Basing on both Ta dependences, the optimal annealing temperature has been set to 310 °C. For Ta = 310 °C the first quarters of B(H) curves are shown in Figure 5a.

In Figure 5b, the real and imaginary parts of magnetic permeability (µ’ and µ”) as a function of frequency for toroidal cores of Fe_85.45−x_Co_x_Cu_0.55_B_14_ alloys annealed at 310 °C have been shown. The most broaden hysteresis loop for Co = 5% in the inset of Figure 5 corresponds to highest coercivity value. The frequency dependent µ’ and µ” permeabilities are gathered in Figure 5b. For binary Co-free alloy µ’ reaches 1600 for f = 10^4^–10^5^ Hz and the maximum value of losses (µ”) is observed at 5 × 10^5^ Hz. For Co = 2.5% alloy small enhance of the µ’ up to 1650 is clearly seen, along with a shift of the µ” maximum at 10^6^ Hz. For higher Co containing alloys, deterioration of soft properties is visible through the decrease of µ’ to the value 700, 550 and 400 for alloys containing 5%, 7.5% and 10% of Co, respectively. The maximum of µ” for these alloys are subsequently shifted into higher frequencies with higher amount of cobalt and are observed at f = 2 × 10^6^ Hz for Co = 5%, f = 4 × 10^6^ Hz for Co = 7.5% and f = 6 × 10^6^ Hz for Co = 10%. From the application point of view, one of the most crucial parameter is core power losses Ps obtained from hysteresis loop at given frequency and magnetic induction. Thus, for heat-treated at optimal conditions, toroidal cores Ps were measured in 50 Hz at 1 T and 1.5 T. Additionally, the saturation magnetization Bs and coercivity Hc have been acquired from the hysteresis loops and all data have been gathered in Table 1. The minimum Ps value measured for 50 Hz/1 T is equal to 0.34 for Co = 2.5% alloy, while for 50 Hz/1.5 T the minimum Ps value is observed for an alloy containig 10% of cobalt.

**Figure 6:**
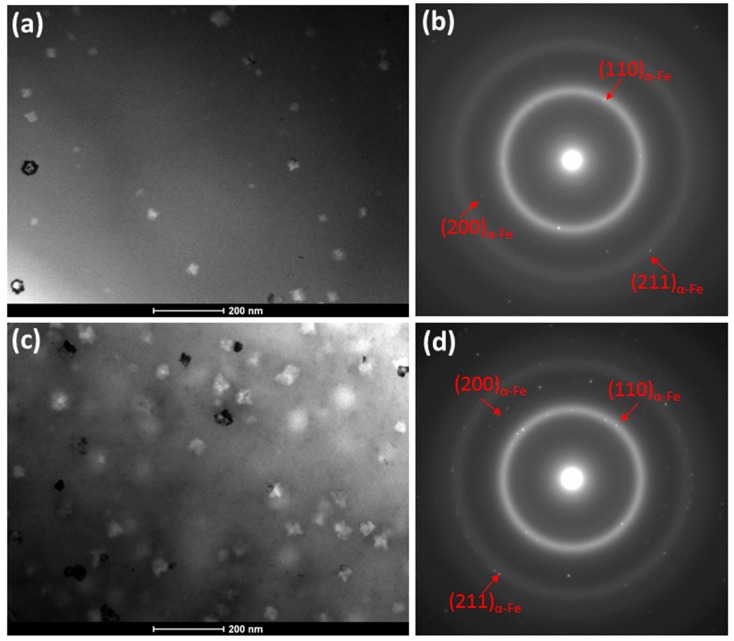
TEM images of annealed at 310 °C samples: (**a**) BF of Fe_85.45_Cu_0.55_B_14_, (**b**) SADP of Fe_85.45_Cu_0.55_B_14_ at 310 °C, (**c**) BF of Fe_75.45_Co_10_Cu_0.55_B_14_, (**d**) SADP of Fe_75.45_Co_10_Cu_0.55_B_14_ at 310 °C.

For the Cu containing alloys, Cu plays a crucial role in the formation of the nanocrystalline state and the existence of soft magnetism is related to the appearance of the nanocrystalline state. Thus, the TEM observations in BF mode (Figure 6a,c) and SADPs (Figure 6b,d) for samples with Co = 0 and Co = 10%, respectively, proved the presence of ~20–30 nm α-Fe nanocrystals. From the TEM observations, it is clearly seen that for annealed Co-free ribbon the number of nanocrystals is much lower then for annealed alloy with 10 wt.% of Co. The amorphous state and nanocrystals coexist and such local atomic arrangement is responsible for optimum magnetic properties.

## 5. Conclusions

The ribbons of nominal composition Fe_85.45−x_Co_x_Cu_0.55_B_14_ show enhancement of the soft magnetic properties with small Co addition (2.5 at.%) with µ’ = 1650 Bs = 1.6 T and P_10/50_ = 0.34 W/kg. The optimal annealing temperature based on Bs(Ta) and Hc(Ta) dependences of all the alloys are in the range 310–320 °C. The alloy with 10% Co addition shows promising saturation induction 1.79 T with still reasonable magnetic core losses of P_10/50_ = 0.37 W/kg, while the soft magnetic properties are deteriorated with µ’ = 400. Additional calorimetric studies revealed that the composition with best soft magnetic properties (Co = 2.5%), is characterized by the maximum of crystallization enthalpy, however the minimum of activation energy of crystallization of α-Fe phase occurs for alloy with Co = 5%. Deeper studies on the atomistic level of this nanocrystallization process should explain this effect.

## Figures and Tables

**Table 1:** Core power losses for annealed at 310 °C toroidal cores measured at 1 T/50 Hz (P10/50), 1.5 T/50 Hz (P15/50), saturation magnetization Bs and coercivity Hc.

**Co content [at.%]**	0	2.5	5	7.5	10
**P_10/50_ [W/kg]**	0.4	0.34	1.1	0.43	0.37
**P_15/50_ [W/kg]**	0.88	0.99	1.9	0.86	0.84
**Bs [T]**	1.56	1.6	1.73	1.77	1.78
**Hc [A/m]**	21.1	16.2	55.8	26.3	23.8

## References

[1] Webster J.G. (2016). Wiley Encyclopedia of Electrical and Electronics Engineering.

[2] Yoshizawa Y., Oguma S., Yamauchi K. (1988). New Fe-based soft magnetic alloys composed of ultrafine grain structure. J. Appl. Phys..

[3] Yoshizawa Y., Yamauchi K., Yamane T., Sugihara H. (1988). Common-mode choke cores using the new Fe-based alloys composed of ultrafine grain structure. J. Appl. Phys..

[4] Willard M.A., Daniil M., Kniping K.E. (2012). Nanocrystalline soft magnetic materials at high temperatures: A perspective. Scr. Mater..

[5] Ohta M., Yoshizawa Y. (2007). New High-Bs Fe-Based Nanocrystalline Soft Magnetic Alloys. Jpn. J. Appl. Phys..

[6] Ohta M., Yoshizawa Y. (2008). Magnetic properties of high-Bs Fe–Cu–Si–B nanocrystalline soft magnetic alloys. J. Magn. Magn. Mater..

[7] Ohnuma M., Hono K., Linderoth S., Pedersen J.S., Yoshizawa Y., Onodera H. (2000). Small-angle neutron scattering and differential scanning calorimetry studies on the copper clustering stage of Fe-Si-B-Nb nanocrystalline alloys. Acta Mater..

[8] Radoń A., Włodarczyk P., Hawełek Ł., Kądziołka-Gaweł M., Gębara P., Nowosielski R., Babilas R. (2018). Thermodynamic approach for determining chemical composition of Fe-Co based amorphous alloys with high thermal stability and glass forming ability. J. Alloys Compd..

[9] Zhang Y., Sharma P., Makino A. (2014). Effects of Cobalt Addition in Nanocrystalline Fe_83.3_Si_4_B_8_P_4_Cu_0.7_ Soft Magnetic Alloy. IEEE Trans. Magn..

[10] Kolano-Burian A., Kolano R., Varga L.K. (2009). Magnetically induced anisotropy in Co rich Finemet type nanocrystalline alloys. J. Alloys Compd..

[11] Kolano-Burian A. (2013). Magnetic domain structure and transverse induced magnetic anisotropy in CoFeCuNbSiB alloys. J. Appl. Phys..

[12] Kolano-Burian A., Kolano R., Hawełek Ł., Szynowski J., Włodarczyk P. (2014). Correlation between nanocrystalline and magnetic structure of Co-based alloys with the induced transverse magnetic anisotropy. J. Appl. Phys..

[13] Kolano-Burian A., Kulik T., Vlasak G., Ferenc J., Varga L.K. (2004). Effect of Co addition on nanocrystallization and soft magnetic properties of (Fe_1−x_Co_x_)_73.5_Cu_1_Nb_3_Si_13.5_B_9_ alloys. J. Magn. Magn. Mat..

[14] Kissinger H.E. (1957). Reaction Kinetics in Differential Thermal Analysis. Anal. Chem..

